# Experience with orthodontic temporary anchorage devices

**DOI:** 10.1186/1746-160X-10-S1-O10

**Published:** 2014-12-12

**Authors:** Marc Schätzle

**Affiliations:** 1 Private Practice, Lucerne, Switzerland

## 

In orthodontics, anchorage is a prerequisite for the application of therapeutic forces and can limit their successful use. According to the intended treatment goals, desired tooth movements should, therefore, be maximized and undesirable effects minimized.

But additional anchorage such as extra- and intraoral forces are visible and hence compliance dependent and are associated with the risk of undesirable effects such as tipping of the occlusal plane, protrusion and gingival recession of mandibular incisors and extrusion of teeth.

Since patients’ cooperation is not always optimal and therefore absolute anchorage is not provided, temporary anchorage devices (TAD) have been introduced. In a recently performed systematic review the survival rate of four different TADs (palatal implants, onplants, miniplates and miniscrews), the palatal implant together with the miniplates has shown the highest survival rate of about 90% and therefore can be considered as a reliable skeletal anchor in the maxilla.

Indications for orthodontic implant anchorage include: Inadequate periodontal anchorage, non-compliant patients for extra- and/or intraoral anchorage aids, prevention of potential side effects of conventional anchorage devices, aesthetic aspects or avoidance of orthognathic surgery after growth completion. Moreover, prosthetic reconstruction may be avoided. The simplicity in use, minimal stress during surgical implant installation and removal as well as reliable success rates are prerequisites for the high acceptance of this treatment by the orthodontic patients. It must be kept in mind, however, the treatment objectives may be achieved by several treatment plans. Proper orthodontic anchorage should be chosen according to the preceding diagnosis and to fit the appropriate treatment plan.

